# Acute toxicity data of common agricultural insecticides to Japanese wild bees

**DOI:** 10.1016/j.dib.2023.108901

**Published:** 2023-01-16

**Authors:** Yugo Seko, Makihiko Ikegami, Tomoyuki Yokoi, Mito Ikemoto, Koichi Goka, Yoshiko Sakamoto

**Affiliations:** a National Institute for Environmental Studies, Onogawa 16-2, Tsukuba, Ibaraki 305-8506, Japan; b Faculty of Life and Environmental Sciences, University of Tsukuba, Tennodai 1-1-1, Tsukuba, Ibaraki 305-8577, Japan

**Keywords:** Ecotoxicology, Pollinator, Topical exposure, Bee, Median lethal dose, Acute exposure, Neonicotinoid, Insecticide

## Abstract

Although numerous ecotoxicological assessments of European honeybee (*Apis mellifera* L.) have been performed, Japanese wild bees are less well studied in this regard. To address this knowledge gap, we assessed the mortality and acute toxicity (LD_50_) of 3 common agricultural insecticides (clothianidin, fipronil, and diazinon) on as many as 6 species of Japanese wild bees (*Andrena prostomias* Perez*, Apis cerana japonica* Radoszkowski*, Bombus deuteronymus* Perez*, Bombus honshuensis* Tkalcu*, Bombus hypocrita* Perez*,* and *Eucera* spp.; all or any of them). The datasets were obtained via standard acute toxicity testing, with assessment of mortality at 24 and 48 h after exposure to the insecticides. These data provide important information regarding the effects of insecticides on Japanese wild bees and their conservation.

**Specifications Table**

**Table:** 

Subject	Biology
Specific subject area	Ecotoxicology
Type of data	Table(s)
How the data were acquired	24 and 48 h acute toxicity tests were performed by visual observation and LD_50_ were estimated by using the “drc” package of R version 4.2.0.
Data format	Raw and analyzed
Description of data collection	Acute toxicity tests to 6 Japanese wild bees on 3 common agricultural insecticides were carried out. Their mortalities were obtained by counting the number of dead bees after 24 and 48 h exposure.
Data source location	Institution: National Institute for Environmental Studies (NIES) City/Town/Region: Onogawa 16-2, Tsukuba city, Ibaraki Prefecture 305-8506 Country: Japan Latitude and longitude: Shown in Table 1.
Data accessibility	Repository name: Mendeley Data Direct URL to data: https://data.mendeley.com/datasets/rwhzf62scn doi:10.17632/rwhzf62scn.3

## Value of the Data

• Honeybees and other wild bees are among the most effective pollinators, providing essential ecosystem services for wild plant reproduction and agriculture production [1,2]. Although data regarding the exposure of honeybees (especially *Apis mellifera*) to insecticides have been accumulating in recent years, that of other Japanese wild bees have been overlooked. Our data provide ecotoxicological data on some of these less-studied species.
• Although the Ministry of the Environment (MoE) in Japan requires ecotoxicological data of wild bees for their conservation [3], few of these data have been collected. Therefore, the acute toxicity data that we have collected for 6 Japanese wild bees likely will be highly beneficial for MoE and conservation ecologists.
• We anticipate that our ecotoxicological assessment of 6 Japanese wild bee species will be used for Species Sensitivity Distribution analysis (SSD) [4] and help to refine the ecotoxicological assessment of pesticides on wild bees in Japan.

## 1 Objective

Although pollinators like bees provide essential ecosystem service for wild plant reproduction and agricultural production [1,2], ecotoxicological data for conservation of these wild bees are lacking in Japan [3]. Our objective is to obtain acute toxicity data for 6 Japanese wild bee species (*Andrena prostomias, Apis cerana japonica, Bombus deuteronymus, Bombus honshuensis, Bombus hypocrita,* and *Eucera* spp.) after exposure to a maximum of 3 common agricultural insecticides (clothianidin, fipronil, and diazinon) in Japan.

## 2 Data Description

Table 1: Details of sampling sites (coordinates), dates, and exposed insecticides of *Andrena prostomias, Apis cerana japonica, Bombus deuteronymus, Bombus honshuensis, Bombus hypocrita,* and *Eucera* spp*.*

**Table 1:** Details of samples.

Species	Sex	Site	Longitude	Latitude	Sampling date	Exposed insecticide (s)
*Andorena prostomias*	Female	Kindai University, Nakamachi 3327-204, Nara city, Nara Prefecture, Japan	34°40′12.6″N	135°44′16.0″E	29-May 2019	Clothianidin, Fipronil
*Apis cerana japonica*	Female (worker)	NIES, Onogawa 16-2, Tsukuba city, Ibaraki Prefecture, Japan	36°02′54.2″N	140°06′54.6″E	19-May 2019	Clothianidin, Fipronil
*Bombus deuteronymus*	Female (worker)	Sugadaira Kogen, Ueda city, Nagano Prefecture, Japan	36°32′13.2″N	138°20′53.1″E	August and September 2019	Clothianidin, Fipronil
*Bombus honshuensis*	Female (worker)	Sugadaira Kogen,Ueda, Nagano Prefecture, Japan	36°32′13.2″N	138°20′53.1″E	August and September 2019	Clothianidin
*Bombus hypocrita*	Female (worker)	Sugadaira Kogen, Ueda city, Nagano Prefecture, Japan	36°32′13.2″N	138°20′53.1″E	August and September 2019	Clothianidin
*Eucera* spp.	Female	University of Tsukuba, Tennodai 1-1-1, Tsukuba city, Ibaraki Prefecture, Japan	36°06′14.5″N	140°06′07.6″E	April and May 2019	Clothianidin, Diazinon, Fipronil

Table 2: Mortality rates of those bee species (some or all of them) at 24 and 48 h after exposure to clothianidin, fipronil, and diazinon.

**Table 2:** Mortality of agricultural insecticides to Japanese wild bees.

					Mortality	
Pesticide	Species	Date	Dose (µg/bee)	Tested Individuals	24 h	48 h
Clothianidin	*Andrena prostomias*	03-06-2019	0	10	0.00	0.00
		03-06-2019	0.002	10	0.20	0.20
		03-06-2019	0.01	10	0.10	0.30
		03-06-2019	0.05	10	1.00	1.00
		03-06-2019	0.25	10	1.00	1.00
	*Apis cerana japonica*	13-06-2019	0	10	0.00	0.00
		13-06-2019	0.0004	10	0.00	0.00
		13-06-2019	0.002	10	0.00	0.00
		13-06-2019	0.005	10	0.40	0.60
		13-06-2019	0.01	10	0.60	0.70
		13-06-2019	0.025	10	1.00	1.00
		13-06-2019	0.05	10	1.00	1.00
	*Bombus deuteronymus*	31-08-2019	0	10	0.00	0.00
		31-08-2019	0.01	10	0.10	0.10
		05-09-2019	0.025	10	0.00	0.00
		31-08-2019	0.05	10	0.90	0.90
		05-09-2019	0.125	10	0.70	0.90
		31-08-2019	0.25	10	0.90	0.90
		31-08-2019	1.25	10	1.00	1.00
	*Bombus honshuensis*	05-08-2019	0	5	0.00	0.00
		31-08-2019	0.01	5	0.00	0.00
		05-09-2019	0.025	5	0.00	0.00
		31-08-2019	0.05	5	0.80	0.80
		05-09-2019	0.125	5	0.40	0.40
		31-08-2019	0.25	5	0.80	1.00
		05-09-2019	0.625	5	1.00	1.00
		31-08-2019	1.25	5	1.00	1.00
	*Bombus hypocrita*	05-09-2019	0	5	0.00	0.00
		05-09-2019	0.002	5	0.00	0.60
		05-09-2019	0.01	5	0.00	0.40
		05-09-2019	0.05	5	0.60	0.60
		05-09-2019	0.25	5	0.80	1.00
	*Eucera* spp.	03-05-2019	0	8	0.00	0.00
		03-05-2019	0.0004	8	0.00	0.00
		03-05-2019	0.002	8	0.00	0.00
		03-05-2019	0.01	8	0.13	0.13
		03-05-2019	0.05	8	0.75	1.00

Diazinon	*Eucera* spp.	17-05-2019	0	10	0.10	0.10
		17-05-2019	0.002	10	0.00	0.10
		17-05-2019	0.01	10	0.10	0.10
		17-05-2019	0.05	10	0.20	0.30
		17-05-2019	0.1	10	0.00	0.10
		17-05-2019	0.25	10	0.00	0.10
		17-05-2019	1.25	10	0.20	0.80

Fipronil	*Andrena prostomias*	03-06-2019	0	10	0.00	0.00
		03-06-2019	0.002	10	0.00	0.10
		03-06-2019	0.01	10	0.10	0.60
		03-06-2019	0.05	10	0.50	1.00
		03-06-2019	0.25	10	0.90	1.00
	*Apis cerana japonica*	17-06-2019	0	10	0.00	0.00
		17-06-2019	0.0004	10	0.00	0.00
		10-09-2019	0.001	10	0.00	0.00
		17-06-2019	0.002	10	0.20	0.20
		10-09-2019	0.005	10	0.90	1.00
		17-06-2019	0.01	10	0.60	0.90
		10-09-2019	0.025	10	1.00	1.00
		17-06-2019	0.05	10	1.00	1.00
		17-06-2019	0.25	10	1.00	1.00
	*Bombus deuteronymus*	05-09-2019	0	10	0.00	0.00
		05-09-2019	0.025	10	0.00	0.60
		05-09-2019	0.05	10	0.40	1.00
		05-09-2019	0.125	10	0.30	0.80
		05-09-2019	0.25	10	1.00	1.00
		05-09-2019	0.625	10	0.70	1.00
	*Eucera* spp.	17-05-2019	0	10	0.10	0.10
		17-05-2019	0.0004	10	0.00	0.00
		17-05-2019	0.002	10	0.10	0.20
		17-05-2019	0.01	10	0.10	0.10
		17-05-2019	0.05	10	0.00	0.70
		17-05-2019	0.25	10	0.20	0.90

“0” in Dose column indicates acetone-only control group.

Table 3: 50% lethal dose (LD_50_) of each insecticide for each bee species as estimated from the mortality data in Table 2.

**Table 3:** LD_50_ values calculated by using the 24 and 48 h mortality data.

			24 h					48 h				
					95% confidence interval					95% confidence interval		

Pesticide	Species	Weight (mean ± sd) (mg)	LD_50_ (µg/bee)	Standard error	Lower	Upper	LD_50_ (µg/mg bee)	LD_50_ (µg/bee)	Standard error	Lower	Upper	LD_50_ (µg/mg bee)
Clothianidin	*Andrena prostomias*	76.07143 ± 13.17223	0.01253	0.18841	NaN	0.82321	0.00016	0.01378	0.00420	NaN	0.03187	0.00018
	*Apis cerana japonica*	77.08873 ± 18.31489	0.00706	0.00075	0.00497	0.00915	0.00009	0.00510	0.00076	0.00300	0.00721	0.00007
	*Bumbus deuteronymus*	172.82813 ± 40.77817	0.04606	0.26217	NaN	0.77133	0.00027	0.04216	0.04232	NaN	0.15966	0.00024
	*Bombus honshuensis*	207.32 ± 58.24216	0.04343	0.03726	NaN	0.16890	0.00021	0.06214	0.03050	NaN	0.14053	0.00030
	*Bumbus hypocrita*	400 ± 89.74651	0.07312	0.01556	NaN	0.11302	0.00042	0.00366	0.00751	NaN	0.03596	0.00002
	*Eucera* spp.	144.95 ± 14.96962	0.02800	0.00033	0.02657	0.02943	0.00019	0.02859	NaN	NaN	NaN	0.00020

Diazinon	*Eucera* spp.	144.95 ± 14.96962	2.31701	NaN	NaN	NaN	0.01598	0.93038	0.90755	NaN	3.45015	0.00642
Fipronil	*Andrena prostomias*	76.07143 ± 13.17223	0.05006	0.00110	0.04533	0.05480	0.00066	0.00775	0.00053	0.00545	0.01005	0.00010
	*Apis cerana japonica*	77.08873 ± 18.31489	0.00328	0.00092	0.00102	0.00554	0.00004	0.00233	0.00134	NaN	0.00561	0.00003
	*Bumbus deuteronymus*	172.82813 ± 40.77817	0.11911	0.05891	NaN	0.30658	0.00069	0.02370	0.00639	0.00335	0.04404	0.00014
	*Eucera* spp.	144.95 ± 14.96962	0.41083	0.01268	0.37048	0.45118	0.00283	0.03849	0.00808	0.01278	0.06421	0.00027

NaN, calculation results in a negative or nonreal number.

The data file containing these tables is available in Mendeley Data [5].

## 3 Experimental Design, Materials, and Methods

### 3.1 Sample Collection

From May through September 2019, workers (sterile females) of *Bombus deuteronymus, Bombus honshuensis*, and *Bombus hypocrita* and females of *Andrena prostomias* and *Eucera* spp. were collected in their natural habitat; collection sites are listed in Table 1. Workers of *Apis cerana japonica* were collected from reared colonies in our laboratory; *A. cerana japonica* brood combs were cut into small pieces and kept in a dark incubator (35°C) to allow adult worker bees to emerge [6]. Emerged workers were maintained at 25°C in an incubator until acute toxicity tests were conducted. As mentioned earlier, we conducted acute toxicity tests by using field-collected adults (except for *A. cerana japonica*). Therefore, differences in the ages (no. of days post-hatching) of bees may have affected their susceptibility to insecticides.

### 3.2 Acute Toxicity Test

We conducted all acute toxicity tests at the same location (NIES). Most (but not all) species were tested on the same day (Table 2). The active ingredients of each insecticide (clothianidin, 99.7%; fipronil, 99.4%; diazinon, 99.4%; FUJIFILM Wako Pure Chemical Corporation) were adjusted to the indicated concentrations (Table 2) by using acetone. Bees were placed individually and housed in plastic cups (φ86 × 40 mm), allowed to acclimate for more than 12 h, and then mildly anesthetized by chilling at 5°C for 15 min before insecticide application. According to OECD test guidelines [7], 2 μL of diluted insecticide solution should be applied to the dorsal thorax of a bee. However, two of the bee species (*Andrena prostomias, Eucera* spp.) we evaluated were too small and hairy to smoothly apply insecticides onto the dorsal thorax. This situation suggests that the evaporation of the acetone is greater in species with smaller body size, making it difficult to ensure uniform insecticide exposure among species. To overcome this problem, we applied 2 μL of diluted insecticide solution to the abdomen of bees, which was easy to accomplish regardless of the bee species. Additional bees were treated with acetone only, as controls. After application, bees were housed in individual plastic cups that had filter paper at the bottom and a 1.5-mL microtube containing a 50% (w/v) solution of sucrose in water as food. Bees were kept under thermostatic conditions of 22°C (light:dark, 16:8) for *Andrena prostomias* and *Eucera* spp. and of 25°C (total darkness) for the other 4 bee species. At 24 and 48 h after insecticide application, plastic cups were briefly removed from the incubator to record the number of bees that had died by visual observation, and mortality rates for each time point and insecticide concentration were calculated (Table 2). When the mortality in the control group (0 in Dose column, Table 2) exceeded 10%, the experimental results were excluded from the data set (i.e., in the *n* = 5 control group, if even one bee died, the experimental results were excluded from the data set). Moreover, mortality of the treatment group was corrected by using Abbott's formula [8],

$$\mathrm{corr}\mathrm{ected}\;\mathrm{mort}\mathrm{ality}\;=\frac{\text{mortality}\;\text{of}\;\text{treatment}\;\text{group}-\text{mortality}\;\text{of}\;\text{control}\;\text{group}}{1-\text{mortality}\;\text{of}\;\text{control}\;\text{group}}$$

and any correction that resulted in a negative calculated value was converted to zero mortality [9]. Note that although we collected enough number of bees for our study, we were unable to obtain mortality and toxicity data for all combinations of 3 insecticides x 6 bee species. Because some of them died during transport to our laboratory and some combinations resulted in higher mortality (> 10%) in the control groups as a result of acute toxicity tests (Table 2). After completion of acute toxicity testing, each tested bee was weighed individually to calculate LD_50_ values on a per-weight basis (see *Statistical analysis*).

### 3.3 Statistical Analysis

The results of acute toxicity tests were fitted by using two-parameter log-logistic regression to estimate the LD_50_ and its 95% confidence interval for each insecticide and species; corrected LD_50_ values were obtained by using the weights of bees (i.e., µg insecticide per mg bee) (Table 3). It should be noted that the mortality data for each bee species used to estimate the LD_50_ all showed low replication, such that the LD_50_ estimation is by no means high quality. These analyses were conducted by using the “drc” package [10] of R version 4.2.0 [11].

## Ethics Statement

This study involved no experimentation on humans or vertebrate animals. These experiments were complied with ARRIVE guidelines and were carried out in accordance with the U.K. Animals (Scientific Procedures) Act, 1986 and associated guidelines.

## CRediT authorship contribution statement

**Yugo Seko:** Writing – original draft, Formal analysis. **Makihiko Ikegami:** Conceptualization, Investigation, Writing – review & editing. **Tomoyuki Yokoi:** Resources, Writing – review & editing. **Mito Ikemoto:** Resources, Writing – review & editing. **Koichi Goka:** Conceptualization. **Yoshiko Sakamoto:** Conceptualization, Writing – review & editing, Investigation, Supervision.

## Declaration of Competing Interest

The authors declare that they have no known competing financial interests or personal relationships that could have appeared to influence the work reported in this paper.

## Data Availability

Acute toxicity data of common agricultural insecticides to Japanese wild bees (Original data) (Mendeley Data).
